# Modeling of methane formation in gravity sewer system: the impact of microorganism and hydraulic condition

**DOI:** 10.1186/s13568-018-0559-6

**Published:** 2018-03-07

**Authors:** Jingwei Xu, Qiang He, Hong Li, Chun Yang, Yinliang Wang, Hainan Ai

**Affiliations:** 1 Chongqing Huantou Environmental Big Data Service & Environmental Engineering Co. Ltd, A1 District, Milan Street, Yubei District, Chongqing, 401123 People’s Republic of China; 2 Key Laboratory of the Three Gorges Reservoir Region’s Eco-Environment, Ministry of Education, Chongqing University, Chongqing, 400045 People’s Republic of China; 3 Transportation Design & Research Sub-Institute, Southwest Municipal Engineering Design & Research Institute of China, Star Road No. 11, Chengdu, Sichuan 610081 People’s Republic of China

**Keywords:** Sewers, Wall-shear stress, Methane production, Microorganism, Model

## Abstract

Sewer system is an important source of methane formation and emission. Although some models were developed to predict methane production in sewers, the impact of microorganism amount was indicated indirectly. Here, seven laboratory scale sewers with varied wall-shear stresses were established. The biofilm thickness, microorganism amount, DO distribution, microorganism community in the biofilms and methane production in the sewers were measured. Based on experimental data, an empirical model was developed to directly describe the relationship between methane production, microorganism amount and wall-shear stress. The results showed that DO concentration decreased significantly along the biofilm depth under varied wall-shear stress, and the DO reduction rate was positively related to the intensity of wall-shear stress. The dominant archaea species in mature biofilms were similar whereas the proportions showed remarkable differences. The abundance of *Methanospirillum* in biofilms cultured at 2.0 Pa wall-shear stress was 53.08% more than that at 1.29 Pa. The maximum methane production rate, 2.04 mg/L wastewater day, was obtained when the wall-shear stress kept at 1.45 Pa, which was 1.2-fold higher than the minimum in sewer at 0.5 Pa. The R^2^ value of the established model was 0.95, the difference between the measurement and simulation was in the rage of 1.5–13.0%.

## Introduction

The control of methane emission have received global considerations. Previous studies were mostly focused on methane emissions from wetlands, termites, ruminants, rice agriculture, fossil fuel exploitation, landfills and biomass burning (Sawakuchi et al. 2016; Weller et al. 2016; Zhao et al. 2016). However, another potential source of methane emission, sewers, was not paid adequate attentions. Methane emission from sewage treatment was found to constitute approximately 5% of the global methane sources (El-Fadel and Massoud 2001). For example, according to National Bureau of Statistics of China, there were 540,000 km sewers in China till 2015 and the average annual growth rate was 7.0% in recent 3 years. In addition, more than 95% of the sewers are gravitational. And a large amount of methane can be produced in gravity sewers and diffused into the atmosphere through manhole. Therefore, controlling methane emission from sewers has a great significance for global climate change.

Recently, some researchers were devoted to studying the reduction of methane in sewers, mainly using chemicals, such as ferric iron (Zhang et al. 2009), nitrate (Jiang et al. 2013) and oxygen injection (Ganigue and Yuan 2014). Although most of these chemicals can inhibit methane production in sewers effectively, they were usually costly and accompanied by some additional problems to subsequent treatment such as increased sludge (Ganigue et al. 2011; Sharma et al. 2012). Methane can be formed in sewers as a result of methanogenic archaea (MA) metabolism (Guisasola et al. 2008). Hydraulic conditions can affect the biofilm’s structure and biological community in sewers (Laspidou and Rittmann 2004; Rochex et al. 2008) hence had a great impact on the formation and emission of methane. This indicated that the methane production could be controlled through manipulation of hydraulic conditions, in an environmental-friendly and cost- effective way. In order to study methane production in sewers quantitatively, previous studies had developed empirical models. Foley et al. (2009) developed an empirical model (Eq. 1) for estimating methane emissions from rising main sewer systems _ENREF_14.

1 $$C_{{CH_{4} }} \, = \,5.24\, \times \,10^{ - 5} \cdot \left[ {\frac{A}{V}\, \times \,HRT} \right]\, + \,0.0015$$

where $C_{{CH_{4} }}$ is the mass CH_4_ emission per unit volume of wastewater (kg/m^3^), A/V is the surface area to volume ratio of sewer, (m^−1)^ and HRT is wastewater retention time in sewer (h).

Despite the fact that the results of model fitted well with field data, the influence of wastewater temperature, which was essential to methane formation, was neglected in the model. Consequently, Chaosakuo et al. (2014) developed another empirical model based on the Foley’s model for predicting CH_4_ emission from gravity-flow sewers located in tropical areas of developing countries (Eq. 2) _ENREF_15.

2 $$C_{{CH_{4} }} \, = \,6\, \times \,10^{ - 5} \cdot 1.05^{{\left( {T - 20} \right)}} \cdot \left[ {\frac{A}{V}\, \times \,HRT} \right]\, + \,0.0015$$

where T is the temperature of sewer wastewater, °C.

In Chaosakuo^’^s model, the wastewater temperature was taken into consideration in comparison to Foley’s model, however, the R^2^ value of model was rather low (0.06). In Chaosakuo^’^s opinion, it may be the limitation of (A/V) HRT, leakages of methane and variation of the climatic conditions that caused the rather low R^2^ value.

The above models were significant to calculate methane production in sewers and both considered the A (surface area of the biofilm), but the effect of microorganism which played the main role in methane formation was overlooked. In fact, methane can be formed as the result of MA metabolism (Guisasola et al. 2008) and maybe it is more reasonable to take into account the amount of microorganism instead of A in gravity sewers.

In this study, the simulated sewers with varied wall-shear stresses were established under laboratory scales. In the simulated sewers, the structure and composition of sewer biofilms were investigated, the DO distribution were evaluated and the methane emission in sewers were assessed as well. The objective of this study is to explain how wall-shear stress affect the methane emission and establish a more solid model to describe or predict methane emission from gravity sewers.

## Materials and methods

### Construction and operation of simulated sewers

**Fig. 1:**
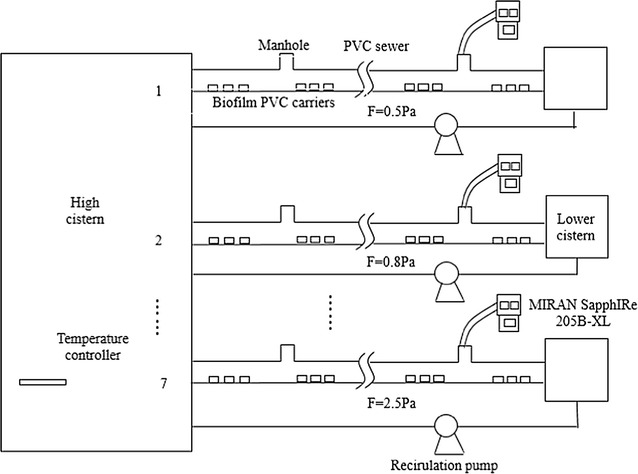
Layout of the pilot gravitational sewers and the sectional view. A well-controlled pilot system was set up to simulate the practical operation of sewers. Seven PVC (Poly Vinyl Chloride) sewers with identical size (with 8.0 m length, 57.0 mm inner diameter and I = 8‰) were placed and operated in parallel under the wall-shear stresses of 0.5, 0.8, 1.12, 1.29, 1.45, 2.0 and 2.5 Pa. Synthetic sewage that added into the seven sewers was prepared in an elevated PVC tank (20.0 L) with the water temperature maintained in the range of 20.0–25.0 °C, which was similar to temperature in real sewers. According to our actual monitoring, the pH was maintained within 7.0–7.5, similar to which was in the real sewer in this study. The outflows were then collected by seven PVC tanks and pumped back to the elevated tank operated 24 h/day. PVC chips (3.0 × 3.0 cm) were installed on the sewer inner-wall below water and they can be removed for regular measurement of thickness of the developed biofilms

A well-controlled pilot system (Fig. 1) was set up to simulate the practical operation of sewers. Seven PVC (Poly Vinyl Chloride) sewers with identical size (with 8.0 m length, 57.0 mm inner diameter and I = 8‰) were placed and operated in parallel under the wall-shear stresses of 0.5, 0.8, 1.12, 1.29, 1.45, 2.0 and 2.5 Pa. The value of wall-shear stress was obtained by combining particle image velocity (PIV) and the Fluent, based on our previous study (Ai et al. 2016). Firstly, the distribution information of the flow field was obtained by PIV. Secondly, the distribution information of the flow field at the same conditions was obtained by modeling used the Fluent and optimizing the parameters. Lastly, the wall shear stress was calculated by the model result of Fluent and the relationship between the shear stress and the factors which affect the flow was established as Eq. 3.

3 $$\begin{aligned} {\text{F}}\, = \, & 0.21941\, + \,0.44146I\, + \,1.73331n\, - \,0.52041\nu \, + \,0.13167nI\, - \,0.24688\nu I \\ & - \,1.47281n\nu \, + \,0.23833I^{2} \, - \,0.12750n^{2} \, + \,1.88828\nu^{2} \\ \end{aligned}$$

where F is the wall-shear stress, Pa; I is the slope of sewers, ‰; n is the fullness degree of sewers; v is the velocity of flow, m/s.

Synthetic sewage that added into the seven sewers was prepared in an elevated PVC tank (20.0 L) with the water temperature maintained in the range of 20.0–25.0 °C, which was similar to temperature in real sewers. According to our actual monitoring, the pH was maintained within 7.0–7.5, similar to which was in the real sewer in this study. And pH in this rage is favorable for the growth of methanogen. The pH was tested every day. According to the daily test results, the pH was regulated using HCl or NaHCO_3_ and maintained at 7.0–7.5. The outflows were then collected by seven PVC tanks and pumped back to the elevated tank operated 24 h/day. PVC chips (3.0 × 3.0 cm) were installed on the sewer inner-wall below water and they can be removed for regular measurement of thickness of the developed biofilms. A synthetic sewage (Glucose: 375 mg/L; NH_4_Cl: 114.6 mg/L; NaH_2_PO_4_·2H_2_O: 50.3 mg/L; MgSO_4_·7H_2_O: 180 mg/L; KCl: 72 mg/L; CaCl_2_: 10.6 mg/L; Peptone: 5 mg/L; NaHCO_3_: 225 mg/L; FeCl_3_·6H_2_O: 375 mg/L; MnCl_2_·4H_2_O: 30 mg/L; H_3_BO_3_: 37.5 mg/L; ZnSO_4_·7H_2_O: 30 mg/L; CuSO_4_·5H_2_O: 7.5 mg/L; EDTA: 30 mg/L; KI: 45 mg/L) was used, according to previous research (Smolders et al. 1994). There were two main reasons for using synthetic sewage. One reason was that it could ignore the effect of inorganic substance when the amount of microorganism was discussed. The another reason was that there was microorganism in real sewage, if the real sewage was used, the portion of methane may be produced by the microorganism in the sewage and it would have an important effect on the methane production.

The experiment was conducted under the initial COD concentration of 400 and 200 mg/L, respectively. The COD in the two group was measured every day and then was supplemented to the initial concentration according to the measured concentration.

### Biofilm characterization

The biofilm thickness and total solids (TS), extracellular polymeric substances (EPS), along with DO distribution in biofilm were assessed in order to evaluate growth of biofilm and determine the time of biofilm maturity (Ai et al. 2016), which could provide a basis for collecting methane and calculating microbial biomass. When the biofilm thickness was stable and the biofilm growth and sloughing were in equilibrium, it was considered to be mature.

### Biofilm thickness

The biofilm thickness was measured with microelectrodes (*Unisense company, Denmark*). The tip diameter was 10 μm. In the process of experiment, the LS18 bracket, microelectrode thruster (MM33-2) and motor controller from *Unisense company* were used. The step distance of μm was achieved by the MM33-2. The electrical signal produced by microelectrode was collected by Microsensor Multimeter and read through the software PRO V.3.1.3 SensorTrace. The oxygen measurement was detailed as follows. In the process of biofilm growth, PVC chips were removed from the sewers to measure biofilm thickness every 5 days. When the micro-electrodes began to get into the biofilm, it began to measure. And when the micro-electrodes started to bend, the measurement was completed. Due to the heterogeneity of the local biofilms, biofilm thickness of nine typical points on each chip was measured and the obtained average value was then regarded as the calculated biofilm thickness.

### Total solids

The chips having been measured for biofilm thickness were placed in ultra-pure water, produced by Simplicity UV (HACH company, USA) and subjected to 40.0 W ultrasound treatment for 1 min at 20 kHz. The pulp was then homogenized and measured by gravimetric method.

#### EPS

The EPS was measured using the method described in our previous research (Xin et al. 2016).

### DO distribution

After the maturation of biofilms, the DO in the biofilms were measured with the DO microelectrode (OX10). Firstly, the DO micro-electrode was connected to the pA channel and polarized, until the stability of signal. Secondly, ran the software STPRO and relevant parameters were set. Thirdly, A standard curve of oxygen concentration was obtained based on oxygen concentration of zero and saturation. Finally, the oxygen in the biofilm was measured with 3-s response time and 100 μm per step. Its response time was less than 3-s and the agitation sensitivity was low.

### Microorganism communities

When anoxic biofilms were fully developed in the pilot sewers, the biofilm samples taken from the chip were then transferred to 2.0 mL plastic centrifuging vials and transported to Sangon Biotech (Shanghai) Co., Ltd for the analysis of microorganism communities using high throughput sequencing (HTS). Plenty of ice cubes were used during sample transportation to avoid the degradation of samples. DNA extraction, PCR amplification and sequencing were all conducted by Sangon Biotech (Shanghai) Co. The composition of the PCR products of 16S rRNA gene was determined by pyrosequencing using the Roche 454 GS-FLX Titanium sequencer (Roche 454 Life Sciences, Branford, CT, USA). Samples in this study were individually barcoded to enable multiplex sequencing. The results are deposited into the NCBI short reads archive database. The accession was Bioproject PRJNA419305 (SRP125386). Details can be seen in our recent articles (Ai et al. 2016).

### Methane production

When anoxic biofilms were fully developed in the pilot sewers after 45 days, the generated methane from the simulated sewer was collected and measured using a Shimadzu GC-9A Gas Chromatograph equipped with an FID once every hour and lasted for 6 days. According to methane concentration every hour and sewers volume, methane production was calculated in sewer every day.

### Model establishing

Based on previous models, a model establishing the relationships between methane emission and microorganism amount, along with methane emission and wall-shear stress in gravity sewers was developed. The model was operated with the experiment results under COD concentration of 400 mg/L and was validated with the experiment results under COD concentration of 200 mg/L.

## Results

In this study, biofilm thickness, density, microbial community and DO distribution were used to describe the sewer biofilm structure, which presented similar trends at seven wall-shear stresses, therefore, the biofilm structures were analyzed and compared at three representative wall-shear stresses (F = 0.8, 1.29 and 2.0 Pa).

### The biofilm growth

**Fig. 2:**
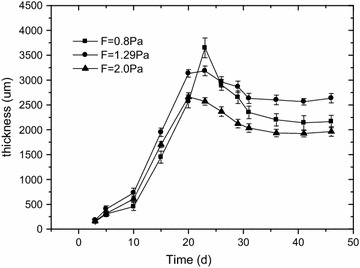
Biofilm thickness versus time at different wall-shear stresses, COD = 400 mg/L (F means wall-shear stress, Pa). At different shear stresses, biofilm thickness changes according to a similar pattern. Firstly, biofilm thickness reached a maximum value within 0–25 days. Then, within 5–10 days after the thickness reaches its maximum value, the thickness of the biofilm decreases to a certain extent. Finally, biofilm thickness tended towards stability. Under the conditions of this experiment, the thicknesses of the biofilms were 2.4 ± 0.1, 2.7 ± 0.1 and 2.2 ± 0.1 mm at shear stresses of 0.8, 1.29 and 2.0 Pa, respectively

**Fig. 3:**
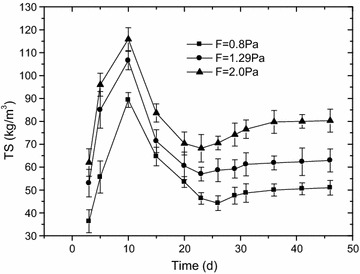
Variation of biomass density at different wall-shear stresses; COD = 400 mg/L (F means wall-shear stress, Pa). At three wall-shear stresses of 0.8, 1.29 and 2.0 Pa, the average biofilm densities were 51 ± 3, 62 ± 5 and 80 ± 5 kg/m^3^, respectively, indicating that the average biofilm densities increased with the increase of wall-shear stress

The biofilm thicknesses and biomass densities variation over time at three shear stress levels were showed on Figs. 2 and 3. At different shear stresses, biofilm thickness changes according to a similar pattern. Firstly, biofilm thickness reached a maximum value within 0–25 days. Then, within 5–10 days after the thickness reaches its maximum value, the thickness of the biofilm decreases to a certain extent. Finally, biofilm thickness tended towards stability. Under the conditions of this experiment, the thicknesses of the biofilms were 2.4 ± 0.1, 2.7 ± 0.1 and 2.2 ± 0.1 mm at shear stresses of 0.8, 1.29 and 2.0 Pa, respectively. Three mature biofilms (biofilm thickness was stable) were obtained after approximately 45 days. At three wall-shear stresses of 0.8, 1.29 and 2.0 Pa, the average biofilm densities were 51 ± 3, 62 ± 5 and 80 ± 5 kg/m^3^, respectively, indicating that the average biofilm densities increased with the increase of wall-shear stress.

### DO distribution in biofilms

**Fig. 4:**
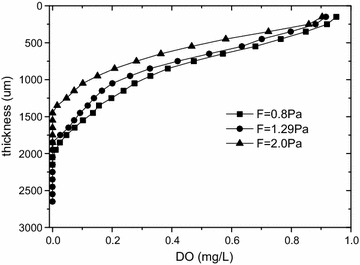
Changes of dissolved oxygen in the biofilm with biofilm thickness, COD = 400 mg/L. The changes of DO along the biofilm depth under three wall-shear stresses were showed. Under three conditions, the DO fell along the biofilm depth and eventually decreased to zero. At the wall-shear stressed of 0.8, 1.29 and 2.0 Pa, the biofilm thicknesses where the dissolved oxygen reduced to zero were 2050, 1850 and 1450 μm, respectively. It revealed that the reduction rate of dissolved oxygen was positively correlated with the wall-shear stress

The changes of DO along the biofilm depth under three wall-shear stresses were showed in Fig. 4. Under three conditions, the DO fell along the biofilm depth and eventually decreased to zero. At the wall-shear stressed of 0.8, 1.29 and 2.0 Pa, the biofilm thicknesses where the dissolved oxygen reduced to zero were 2050, 1850 and 1450 μm, respectively. It revealed that the reduction rate of dissolved oxygen was positively correlated with the wall-shear stress.

### The archaea

The sequences of biofilms at three wall-shear stresses (0.8, 1.29, 2.0 Pa)were 19,326, 17,848 and 9458, the operational taxonomic units (OTUs) were 52, 20 and 10, respectively. The coverage of biofilm samples were all 99.9%, indicating that the results were reasonable and could reflect the microbial community structure of biofilm samples.

**Fig. 5:**
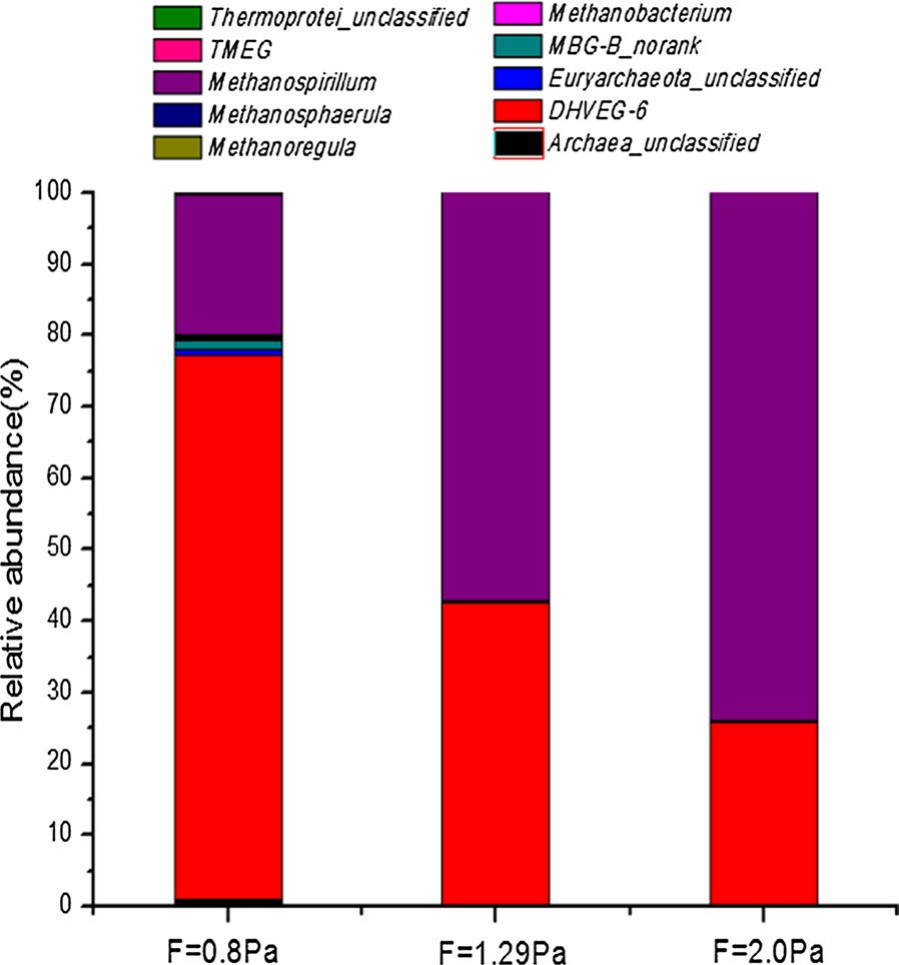
The relative abundance of predominant bacterial phylum in mature biofilms, COD = 400 mg/L. Nine classified *archaea* were detected and listed in Table 1. *Methanospirillum* and *DHVEG*-*6* were both dominant in three biofilms. They both accounted for 96.02, 99.6 and 99.6%, respectively, at wall-shear stresses of 0.8, 1.29 and 2.0 Pa. The proportion of *methanospirillum* in this study increased with the increase of wall-shear stress, but *DHVEG*-*6* was decreased with the increase of wall-shear stress

Nine classified *archaea* were detected and listed in Fig. 5 and Table 1. *Methanospirillum* and *DHVEG*-*6* were both dominant in three biofilms. They both accounted for 96.02, 99.6 and 99.6%, respectively, at wall-shear stresses of 0.8, 1.29 and 2.0 Pa. The proportion of *methanospirillum* in this study increased with the increase of wall-shear stress, but *DHVEG*-*6* was decreased with the increase of wall-shear stress.

**Table 1:** The relative abundance of predominant bacterial phylum in mature biofilms, COD = 400 mg/L

	F = 0.8 Pa	F = 1.29 Pa	F = 2.0 Pa
*Archaea_unclassified (%)*	0.97	0.02	0.01
*DHVEG*-*6 (%)*	76.15	42.53	25.76
*Euryarchaeota_unclassified (%)*	0.89	0.09	0.01
*MBG*-*B_norank (%)*	1.25	0.23	0.03
*Methanobacterium (%)*	0.26	0.02	0
*Methanoregula (%)*	0.4	0.04	0.32
*Methanosphaerula (%)*	0.04	0	0.01
*Methanospirillum (%)*	19.87	57.07	78.84
*TMEG (%)*	0.06	0	0.02
*Thermoprotei_unclassified (%)*	0.11	0	0

### Laboratory-scale methane production

**Fig. 6:**
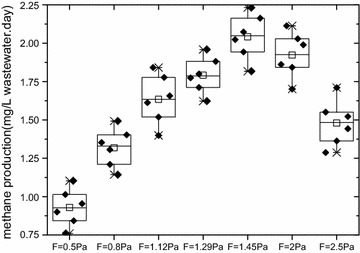
Methane production at different wall-shear stresses, COD = 400 mg/L. The methane production was shown. At seven wall-shear stresses, the average methane production were 0.93, 1.32, 1.63, 1.80, 2.04, 1.93 and 1.48 mg/(L wastewater day), respectively. Methane production increased with the increase of wall-shear stress until the wall-shear stress reached 1.45 Pa. The maximum methane production was 2.04 mg/(L wastewater day) and the minimum methane production was 0.93 mg/(L wastewater day)

The methane production was shown in Fig. 6. At seven wall-shear stresses, the average methane production were 0.93, 1.32, 1.63, 1.80, 2.04, 1.93 and 1.48 mg/(L wastewater day), respectively. Methane production increased with the increase of wall-shear stress until the wall-shear stress reached 1.45 Pa. The maximum methane production was 2.04 mg/(L wastewater day) and the minimum methane production was 0.93 mg/(L wastewater day).

## Discussion

### The effect of wall-shear stress on biofilm physical structure

Previous works have reported that higher shearing stresses led to thinner biofilms (Kwok et al. 1998; Laspidou and Rittmann 2004; Liu and Tay 2002). However, as shown in Fig. 2, the biofilm thickness obtained in this study increased from 2.1 to 2.7 mm while the wall-shear stress increased 0.8 and 1.29 Pa. This may because the range of wall-shear stresses and the structure of pilot sewers were different from which used in the previous research. In the study of Liu and Tay, the shear stresses were 6.5–9.0 Pa. In the study of Kwok, the biofilms were formed in 3-L internal loop airlift reactors. However, our results were supported by the study of Guzmán et al. (2007) which has demonstrated that the wall-shear stress within the range of 1.1–1.4 Pa was suitable for biofilm growth in sewers_ENREF_19. Biofilm density was impacted by some factors including hydraulic condition and microbial species (Christensen and Characklis 1990) (Laspidou and Rittmann 2004). The wall-shear stress affects the biofilms horizontally and vertically. The loose surface of biofilm would be washed away and the attached biofilms were compressed by the increase of wall-shear stress, leading to the increase of the biomass density (Xu et al. 2016). The higher density observed at higher wall-shear stress in this study was supported by others’ research (Liu and Tay 2002; Vieira et al. 1993), that a higher wall-shear stress resulted in a denser biofilm was founded in their studies.

Porosity and density of biofilm are both important to the transformation in biofilm. According to our previous research, the oxygen penetration depth in biofilm was higher in lower wall-shear stress (Xu et al. 2016). The greater wall-shear stress was, the greater the density of biofilm was. In other words, a denser biofilm under higher shear stress could lead to the decreased oxygen penetration depth. Previous studies have shown that slight shearing stress was favorable for the formation of the inattentive and porous structure of biofilm (van Loosdrecht et al. 1995, 2002). Because biofilm porosity decreased with the increase of wall-shear stress, the dissolved oxygen was minimum in the biofilm cultured at wall-shear stress of 2.0 Pa among these three wall-shear stresses. Oxygen played a significant role in the process of microbial growth and the different oxygen conditions inevitably had a major impact on the microbial community structure of the biofilms.

The effect of wall-shear stress on biofilm structure was in three aspects. Firstly, with the increase of wall-shear stress, biofilm became thinner. Then, with the increase of wall-shear stress, biofilm density became greater. Lastly, the microbial community in biofilm was affected by wall-shear stress (Cheng et al. 1997).

### The effect of wall-shear stress on biofilm microbial structure

#### The microorganism amount

**Fig. 7:**
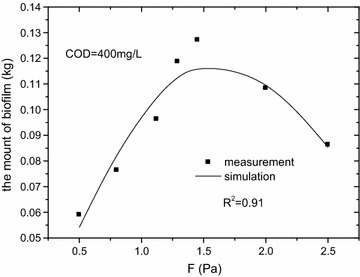
Microorganism amount vs wall-shear stress, COD = 400 mg/L. The amount of microorganism increased with the increase of wall-shear stress until the wall-shear stress reached 1.45 Pa. When wall-shear stress exceeded 1.45 Pa, although the biofilm density increased as well, the thickness of biofilm decreased at a greater degree, resulting the drop of the microorganism amount. When the wall-shear stress was 1.45 Pa, the average biofilm density was 74 ± 5 kg/m^3^, just 7.5% less than which obtained at wall-shear stress of 2.0 Pa, while the biofilm thickness was 2.4 ± 0.1 mm, 20.8% greater than which reached at wall-shear stress of 2.0 Pa. The R^2^ of Eq. (3) was 0.91, indicating that it is reasonable

Biofilm is a complex micro-ecological structure composed of microorganisms and EPS. Flow rate, the diameter of sewer (Guzmán et al. 2007) and substrate concentration were important factors affecting biofilm growth in sewers. In this study, wall-shear stress was calculated by combining flow rate, slope and fullness, which can be seen as a comprehensive factor. Due to the use of synthetic sewage, the inorganic materials in the biofilms were few thus could be ignored. The average mass density of the biofilm (TS) which did not consist of Extracellular Polymeric Substances (EPS) could represent the microorganism quantity. The amount of microorganism was calculated according to the average biofilm density, thickness, the surface area of biofilm growth and the EPS. As show in Fig. 7, the amount of microorganism increased with the increase of wall-shear stress until the wall-shear stress reached 1.45 Pa. When wall-shear stress exceeded 1.45 Pa, although the biofilm density increased as well, the thickness of biofilm decreased at a greater degree, resulting the drop of the microorganism amount. When the wall-shear stress was 1.45 Pa, the average biofilm density was 74 ± 5 kg/m^3^, just 7.5% less than which obtained at wall-shear stress of 2.0 Pa, while the biofilm thickness was 2.4 ± 0.1 mm, 20.8% greater than which reached at wall-shear stress of 2.0 Pa.

According to the calculated data, a simple empirical model (Eq. 4) about microorganism and wall-shear stress was developed

4 $${\text{X}}\, = \, - \,0.0485F^{2} \, + \,0.161F\, - \,0.0142$$

where X is the amount of microorganism (kg); F is wall-shear stress (Pa).

**Fig. 8:**
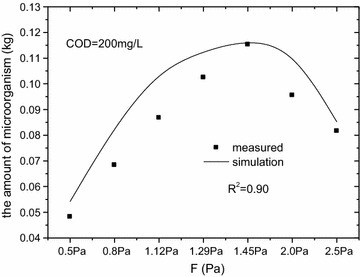
Microorganism amount vs wall-shear stress, COD = 200 mg/L. In addition, in order to validate the model, the measured data was used when COD was 200 mg/L. The R^2^ was 0.90

As shown in Fig. 7, the R^2^ of Eq. (4) was 0.91, indicating that it is reasonable. In addition, in order to validate the model, the measured data was used when COD was 200 mg/L. As the Fig. 8 shown, the R^2^ was 0.90.

#### The effect of wall-shear stress on microbial composition

The wall-shear stress influenced the mass transfer in biofilm and it played an important role in the microbial composition of the biofilm. Previous research was mostly focused on the influence of wall-shear stress on the physical structure such as biofilm thickness and biofilm density (Kwok et al. 1998; Liu and Tay 2002). However, the impact of wall-shear stress on the biofilm microbial composition did not obtain enough attention (Rochex et al. 2008). As shown in Fig. 5, although *Deep*-*Sea*-*Hydrothermal*-*Vent*-*Gp*-*6 (DHVEG*-*6)*-*norank6* and *Methanospirillum* were the dominant bacteria in three biofilms, their proportions were different. *DHVEG*-*6* is known as *haloarchaea*, distantly related to *halobacteriales,* (Casamayor et al. 2013) and has been detected in marine environments, terrestrial soils and saline lakes including deep sea methane seep sediments (Nunoura et al. 2011). The distribution of *DHVEG*-*6* indicated that it could produce methane in this study, though the physiological and metabolic functions of *DHVEG*-*6* were not fully known (Kuroda et al. 2014). *Methanospirillum* was a fastidious anaerobic specie and could produce methane with H_2_–CO_2_, and its preferred living temperature and pH were 30.0–37.0 °C and 6.6–7.4, respectively (Ferry et al. 1974).

In fact, the proportion of *Methanospirillum* in sewer biofilm increased with the increase of wall-shear stress, but the amount was not, because that the microorganism amount did not increase with the increase of wall-shear stress. Although the proportion of *Methanospirillum* in sewer biofilm cultured at wall-shear stress of 1.45 Pa was not the highest, the amount of *Methanospirillum* was at the most. To be exact, the more *Methanospirillum* was, the greater methane production was and it implied that *Methanospirillum* played the crucial role in methane formation in gravity sewers in this study.

### Model development and validation

Methane production is related directly to the amount of microorganism which is influenced by wall-shear stress in sewers. So the wall-shear stress could play an important role in methane production. The aims of model was to make certain of the role that wall-shear stress played in methane production (Ai et al. 2016; Chaosakul et al. 2014).

4 $${\text{X}}\, = \, - \,0.0485F^{2} \, + \,0.161F\, - \,0.0142$$

5 $$Q_{{CH_{4} }} \, = \,Y_{{CH_{4} /X}} \cdot X \cdot {\theta }^{T - 20} \cdot HRT .$$

3 $$\begin{aligned} {\text{F}}\, = \, & 0.21941\, + \,0.44146{\text{I}}\, + \,1.73331{\text{n}}\, - \,0.52041{\text{v}}\, + \,0.13167{\text{nI}} \\ & - \,0.24688{\text{vI}}\, - \,1.47281{\text{nv}}\, + \,0.23833I^{2} \, - \,0.12750n^{2} \, + \,1.88828\nu^{2} \\ \end{aligned}$$

where $Q_{{CH_{4} }}$ is the methane production, mg/(L wastewater day); $Y_{{CH_{4} /X}}$ is the yield coefficient, mg methane/kg biomass; X is the amount of microorganism, kg; $\theta$ is the temperature coefficient = 1.05; T is the temperature of sewer wastewater, °C; HRT is the wastewater retention time, h; F is the wall-shear stress, Pa; I is the slope of sewers, ‰; n is the fullness degree of sewers; v is the velocity of flow, m/s.

Equation (5) was similar to the model previously proposed by Chaosakul et al. (2014) except that the X and $Y_{{CH_{4} /X}}$ took the place of (A/V) and γ (the specific rate of CH_4_ emission), respectively and Eq. (3) was based on our previous study (Ai et al. 2016).

**Fig. 9:**
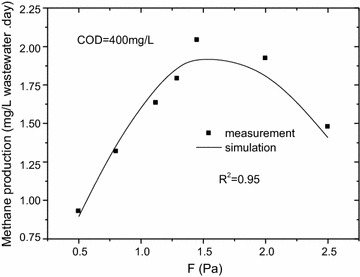
Methane production vs wall-shear stress, COD = 400 mg/L. The relationship between the methane production and wall-shear stress. The R^2^ value of Eq. (5) was 0.95, indicating that the Eq. (5) was also reasonable

Figure 9 illustrated the relationship between the methane production and wall-shear stress. The R^2^ value of Eq. (5) was 0.95, indicating that the Eq. (5) was also reasonable.

**Fig. 10:**
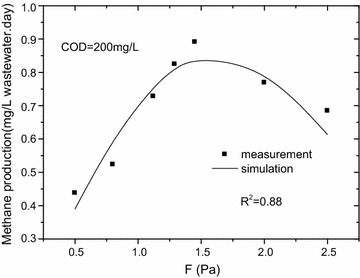
Methane production vs wall-shear stress, COD = 200 mg/L. In order to validate the model, the measured data was used when COD was 200 mg/L. The fitting results were showed. Results showed the model’s predictions, agreed with the measurements well, the difference between measurement and simulation was found in the rage of 1.5–13.0%. In this model, substrate concentration and pipe size were not considered that leaded to some errors between measurement and simulation. And it would be revised in our future research

In order to validate the model, the measured data was used when COD was 200 mg/L. The fitting results were showed in Fig. 10. Results showed the model’s predictions, agreed with the measurements well, the difference between measurement and simulation was found in the rage of 1.5–13.0%. In this model, substrate concentration and pipe size were not considered that leaded to some errors between measurement and simulation. And it would be revised in our future research.

Most of methanogen was strictly anaerobic and a small amount of methanogen was anoxic and aerobic. According to the laying of gravity sewer, there are manholes among the sewers. Usually, there is oxygen in gravity sewers. In this study, the methane production was less than which obtained in rising main sewers in previous research (Guisasola et al. 2008, 2009). An important reason may be that anaerobic environment in rising main sewers is more beneficial to methane production. In addition, rising main sewers were full of sewage so that biofilm could develop on the entire surface of sewers. There is an obvious difference between gravity sewer and rising main sewer. Gravity sewers are not full of sewage. The biofilm could not grow on the places which are not covered with sewage. This would lead to difference in microorganism amount and that affects the methane production.
